# Severe maternal morbidity and near misses in tertiary hospitals, Kelantan, Malaysia: a cross-sectional study

**DOI:** 10.1186/s12889-016-2895-2

**Published:** 2016-03-05

**Authors:** Mohd Noor Norhayati, Nik Hussain Nik Hazlina, Zaharah Sulaiman, Mohd Yacob Azman

**Affiliations:** Women’s Health Development Unit, School of Medical Sciences, Universiti Sains Malaysia, Health Campus, 16150 Kubang Kerian, Kelantan Malaysia; Medical Division, Kelantan State Health Department, Level 2, Wisma Persukutuan, Jalan Bayam, 15590 Kota Bharu, Kelantan Malaysia

**Keywords:** Severe maternal morbidity, Maternal near miss, Maternal deaths, Obstetric complications, WHO near miss approach

## Abstract

**Background:**

Severe maternal conditions have increasingly been used as alternative measurements of the quality of maternal care and as alternative strategies to reduce maternal mortality. We aimed to study severe maternal morbidity and maternal near miss among women in two tertiary hospitals in Kota Bharu, Kelantan, Malaysia.

**Methods:**

A cross-sectional study with record review was conducted in 2014. Severe maternal morbidity and maternal near miss were classified using the new World Health Organization criteria. Health indicators for obstetric care were calculated and descriptive analyses were performed using SPSS version 22.0.

**Results:**

In total, 21,579 live births, 395 women with severe maternal morbidity, 47 women with maternal near miss and two maternal deaths were analysed. The severe maternal morbidity incidence ratio was 18.3 per 1000 live births and the maternal near miss incidence ratio was 2.2 per 1000 live births. The maternal near miss mortality ratio was 23.5 and the mortality index was 4.1 %. The process indicators for essential interventions were almost 100.0 %. Haemorrhagic disorders were the most common event for severe maternal morbidity (68.6 %) and maternal near miss (80.9 %) and management-based criteria accounted for 85.1 %.

**Conclusions:**

Comprehensive emergency care and intensive care as well as overall improvements in the quality of maternal health care need to be achieved to substantial reduce maternal death.

## Background

Investing in maternal health is considered one of the crucial elements in the development agenda of countries. Of the eight Millennium Development Goals (MDGs), goal five includes 'improving maternal health'; this goal consists of two targets, one of which is to reduce maternal mortality by three-quarters between 1990 and 2015. In developing countries, the unacceptably high maternal death overshadows maternal morbidity. Because maternal morbidities occur more frequently than maternal deaths, maternal near miss was suggested as a more useful indicator for the evaluation and improvement of maternal health services than the maternal mortality ratio (MMR) [1].

In Malaysia, the national MMR have shown an impressive decline of 94 % from 530 per 100,000 live births in 1950 to 28 per 100,000 live births in 2009. This decline was largely due to the introduction of competency-based training and placement of midwives in rural areas, in addition to advances in medicine and technology, improvements in the health care delivery system and implementation of a risk approach strategy and confidential enquiry into maternal deaths (CEMD). In spite of these improvements, the MMR has remained plateaued at approximately 28 to 30 per 100,000 live births since the year 2000 [2].

The World Health Organization (WHO) Working Group on Maternal Mortality Morbidity Classifications has recently developed a standard definition and internationally accepted identification criteria for very severe and severe maternal morbidity cases. Hence, maternal near miss or very severe maternal morbidity, is defined as 'a woman who nearly died but survived a complication that occurred during pregnancy, childbirth or within 42 days of termination of pregnancy'. Additionally, severe maternal morbidity refers to ‘potentially life-threatening conditions during pregnancy, childbirth or after termination of pregnancy from which maternal near miss cases would emerge'. The identification of maternal near miss cases is based on the presence of 25 criteria regarding organ and system dysfunction (cardiovascular, respiratory, renal, coagulation, hepatic, neurologic and uterine) via clinical-, laboratory- and management-based parameters. In contrast, the identification of severe maternal morbidity is based on a list of potentially life-threatening conditions from which maternal near miss cases would emerge [3, 4].

Reporting the magnitude of severe and very severe maternal morbidities is the first important step in measuring the quality of a maternity system. This reporting may act as a complement to CEMD or as an alternative strategy to reduce maternal mortality. Second, identifying the severe maternal morbidities or potentially life-threatening conditions from which maternal near miss cases would emerge based on the recently developed standard definition and internationally accepted identification criteria by the WHO [3] allows for valid comparisons across countries and regions. This standardization is important because existing studies showed variation in the definition and criteria used.

This study aims to study severe maternal morbidity and maternal near miss among women as proposed by the WHO near miss approach and related indicators in tertiary hospitals in Kelantan, Malaysia. To our knowledge, this study is the first in Malaysia to assess maternal morbidities using the working guidelines.

## Methods

In a cross-sectional study conducted in Raja Perempuan Zainab II Hospital and Universiti Sains Malaysia Hospital, Kelantan, data from postpartum women were obtained throughout the 1 year period of 2014. Raja Perempuan Zainab II Hospital and Universiti Sains Malaysia Hospital are the two referral and tertiary hospitals with approximately 14,000 and 7000 deliveries per year, respectively [5]. The study population includes all postpartum women regardless of age. Women who developed complications beyond 42 days of termination of pregnancy were excluded. Data regarding the total number of live births and maternal deaths occurring in the facilities during the study period were also collected.

Sample size calculation to determine the prevalence of severe maternal morbidity and maternal near miss was performed using a single proportion formula [6]. The 17.46 % [7] and 2.93 % [8] prevalence, respectively, of severe maternal morbidity and maternal near miss based on the WHO criteria were taken because they yielded the largest sample size. For severe maternal morbidity, taking the precision of 0.01 with 95 % confidence, the minimum required sample size was 5537. After considering a non-response rate of 20 %, the calculated sample size was 6644 postpartum women. For maternal near miss, taking the precision of 0.01 with 95 % confidence, the minimum required sample size was 1093. After considering a non-response rate of 20 %, the calculated sample size was 1312 postpartum women. Therefore, data from a minimum of 6644 women were needed.

However, in this context, obtaining the frequency of severe maternal morbidity and maternal near miss cases for a 1 year period is far more important than estimating the sampling frame for determining prevalence. This overall picture produces a more valid comparison between the severe maternal morbidity ratio, maternal near miss ratio and maternal mortality ratio. Therefore, an approximately 20,000 women at the two facilities in 2014 formed the sampling frame for this study.

The WHO near miss approach was utilized to obtained information regarding morbidity criteria for severe maternal morbidity (haemorrhagic disorders, hypertensive disorders, other systemic disorders and severe management indicators) and organ dysfunction criteria for maternal near miss (vital organ dysfunction or failure, for example, circulatory, respiratory, cardiac, renal, hepatic, central nervous, metabolic and haematological). In addition, care based on essential interventions and its process indicators, i.e., prevention and treatment of postpartum haemorrhage, treatment of eclampsia and prevention and treatment of infection or sepsis, were assessed [4]. Hospital- and home-based medical records were reviewed to retrieve patient information and severe maternal morbidity and maternal near miss criteria. The extracted information included sociodemographic characteristics, current obstetric history, clinical parameters, past obstetric history, medical and gynaecological history, foetal outcome and health care provision.

Severe maternal morbidity and maternal near miss cases were identified throughout the 1 year period at both facilities. A research assistant trained in nursing reviewed the admission registers and medical records in delivery rooms and obstetrics and gynaecology wards daily. Information regarding women with severe maternal morbidity as identified by the life-threatening conditions was obtained. Among these women, maternal near miss cases were identified by the presence of organ system dysfunction. To decrease the risk of selection bias and to minimize the number of missed cases, all pregnancies and deliveries with any medical problem, not only potentially life-threatening conditions, were reviewed. The medical staffs were also asked regarding any cases fulfilling the criteria. The researcher made the final choice for inclusion.

The data were entered and analysed using IBM SPSS Statistics version 22.0 (SPSS Inc., 2013). The data were checked and filtered before analysis. Descriptive analysis was used to determine the prevalence of severe maternal morbidity and maternal near miss based on the denominator of live births. Background characteristics of the women, morbidity and organ dysfunction criteria and process indicators were explored.

This study protocol was approved and the access to patient medical records were issued by the Human Research Ethics Committee, Universiti Sains Malaysia (USM/PPSP®/2012/JKP-62[62.3(4)]) and Medical Research Ethics Committee, Ministry of Health (KKM/NIHSEC/800-2/2/2/Jld 2 P13-215). The data were obtained from the medical records; therefore, this part of the study was exempt from informed consent procedures. The confidentiality of the data regarding the participating women was preserved.

## Results

During the 1 year data collection period, 21,756 deliveries, 21,579 live births, 395 women with severe maternal morbidity, 47 women with maternal near miss and two maternal deaths were recorded. On average, seven to eight women with severe maternal morbidity were identified per week. In total, 308 (78.0 %) women with severe maternal morbidity and 42 (89.4 %) women with maternal near miss delivered at Raja Perempuan Zainab II Hospital. Two maternal deaths were reported.

The severe maternal morbidity incidence ratio was 18.3 per 1000 live births and the maternal near miss incidence ratio was 2.2 per 1000 live births. These ratios were used to calculate the prevalence values of severe maternal morbidity and maternal near miss of 1.83 and 0.22 %, respectively. The sociodemographic and medical characteristics of women with severe maternal morbidity (*n* = 395) and maternal near miss (*n* = 47) are shown in Tables 1 and 2, respectively.

**Table 1:** Sociodemographic characteristics of women with severe maternal morbidity and maternal near miss

Variables	SMM (*n* = 395)	MNM (*n* = 47)
	*n* (%)	*n* (%)
Age (years)	31.5 (6.35)^a^	33.2 (6.03)^a^
Age		
< 35 years	265 (67.1)	27 (57.4)
≥ 35 years	130 (32.9)	20 (42.6)
Age at marriage (years)	23.2 (4.47)^a,b^	24.4 (5.57)^a,h^
Duration of marriage (years)	8.4 (6.98)^a,b^	7 (15.0)^d,h^
Household income (MR/monthly)	1800 (2000.0)^c,d^	2350 (3525.0)^d,e^
Race		
Malay	386 (97.7)	46 (97.9)
Others	9 (2.3)	1 (2.1)
Level of education		
Nil and primary	21 (5.3)	6 (12.8)
Secondary	239 (60.5)	22 (46.8)
Tertiary	135 (34.2)	19 (40.4)
Occupation		
Unemployed	210 (53.2)	19 (40.4)
Self-employed	31 (7.8)	4 (8.5)
Support group	99 (25.1)	13 (27.7)
Professional group	55 (13.9)	11 (23.4)
Marital status		
Married	390 (98.7)	46 (97.9)
Single	5 (1.3)	1 (2.1)
Husband occupation		
Unemployed	7 (1.8)^b^	1 (2.2)^e^
Self-employed	150 (38.6)	19 (41.3)
Non-professional group	188 (48.3)	18 (39.1)
Professional group	44 (11.3)	8 (17.4)

*SMM* severe maternal morbidity, *MNM* maternal near miss

^a^mean (standard deviation)

^b^ *n* = 389. Six women (four singles and two non-Malaysians) have no related marital information

^c^ *n* = 378

^d^median (interquartile range). Skewed to the right

^e^ *n* = 46. One woman unmarried

**Table 2:** Medical characteristics of women with severe maternal morbidity and maternal near miss

Variables	SMM (*n* = 395)	MNM (*n* = 47)
	*n* (%)	*n* (%)
Current obstetric history		
Parity	2 (3.0)^b^	3.0 (3.0)^b^
Booking		
Early (≤ 12 weeks)	196 (53.4)^c^	21 (48.8)^d^
Late (> 12 weeks)	171 (46.6)	22 (51.2)
Antenatal care visits		
Optimum (≥8 visits)	320 (87.2)^c^	37 (86.0)^d^
Suboptimum (<7 visits)	47 (12.8)	6 (14.0)
Hospital stay (day)	4 (2.0)^b^	7 (3.0)^b^
Clinical parameters		
Body mass index at booking (kg/m^2^)		
Normal (18.50–24.99)	118 (32.2)^c^	18 (41.9)^d^
Underweight (≤18.49)	24 (6.5)	3 (7.0)
Overweight (25.00–29.99)	111 (30.2)	8 (18.6)
Obese (≥30.00)	114 (31.1)	14 (32.6)
Systolic blood pressure (mmHg)	134.1 (22.47)^a^	133.9 (23.70)^a^
Diastolic blood pressure (mmHg)	81.0 (13.84)^a^	79.2 (14.16)^a^
Hemoglobin level (g/dl)	11.4 (1.16)^a^	11.1 (1.29)^a^
Past obstetric history		
History of previous caesarean delivery		
Absent	306 (77.5)	30 (63.8)
Present	89 (22.5)	17 (36.2)
History of pregnancy complications		
Absent	306 (77.5)	31 (66.0)
Present	89 (22.5)	16 (34.0)
History of previous abortion		
Absent	310 (78.5)	35 (74.5)
Present	85 (21.5)	12 (25.5)
Medical history		
Comorbidity		
Absent	346 (87.6)	38 (80.9)
Present	49 (12.4)	9 (19.1)

*SMM* severe maternal morbidity, *MNM* maternal near miss

^a^mean (standard deviation)

^b^median (interquartile range). Skewed to the right

^c^ *n* = 367. 28 women were with early pregnancy had no antenatal care follow up

^d^ *n* = 43. Four women were with early pregnancy had no antenatal care follow up

### Morbidity criteria

Table 3 covers the morbidities justifying the inclusion of the affected women in the study. Scrutiny of the data in this table will allow identification of the pattern of morbidities among those women who survived severe pregnancy-related complications. Among women with severe maternal morbidity, haemorrhagic disorders (68.6 %) were the most common criteria for morbidity followed by severe management indicators (54.4 %) and hypertensive disorders (33.4 %). In total, 48.9 and 17.0 % of the cases required blood transfusion and intensive care unit admission, respectively. Among the 395 cases, 183 (46.3 %) women developed severe morbidity conditions at arrival or within 12 h of arrival and 166 (42.0 %) developed severe morbidity conditions 12 h after arrival; of these women, the number of referred cases were 125 (68.3 %) within 12 h of arrival and 115 (69.3 %) 12 h after arrival.

**Table 3:** Morbidity conditions of women with severe maternal morbidity and maternal near miss

Morbidity criteria	SMM (*n* = 395)	MNM (*n* = 47)
	*n* (%)	*n* (%)
Haemorrhagic disorders	271 (68.6)	38 (80.9)
Abruptio placenta	28 (7.1)	3 (6.4)
Placenta accreta/increta/percreta	13 (3.3)	10 (21.3)
Ectopic pregnancy	31 (7.8)	4 (8.5)
Postpartum haemorrhage	210 (53.2)	26 (55.3)
Ruptured uterus	3 (0.8)	2 (4.3)
Hypertensive disorders	132 (33.4)	10 (21.3)
Severe pre-eclampsia	103 (26.1)	7 (14.9)
Eclampsia	22 (5.6)	2 (4.3)
Severe hypertension	11 (2.8)	1 (2.1)
Hypertensive encephalopathy	0 (0)	0 (0)
HELLP syndrome	16 (4.1)	6 (12.8)
Other systemic disorders	56 (14.2)	18 (38.3)
Endometritis	0 (0)	0 (0)
Pulmonary oedema	10 (2.5)	3 (6.4)
Seizures	19 (4.8)	2 (4.3)
Sepsis	2 (0.5)	0 (0)
Shock	9 (2.3)	6 (12.8)
Thrombocytopenia	28 (7.1)	13 (27.7)
Thyroid crisis	0 (0)	0 (0)
Severe management indicators	215 (54.4)	43 (91.5)
Blood transfusion	193 (48.9)	39 (83.0)
Central venous access	2 (0.5)	1 (2.1)
Hysterectomy	19 (4.8)	19 (40.4)
Intensive care unit admission	67 (17.0)	34 (72.3)
Prolonged hospital stay	6 (1.5)	3 (6.4)
Intubation not related to anesthesia	14 (3.5)	9 (19.1)
Returned to operation room	7 (1.8)	7 (14.9)
Laparotomy	22 (5.6)	11 (23.4)

*SMM* severe maternal morbidity, *MNM* maternal near miss

Among women with maternal near miss, severe management indicators (91.5 %) were the most common criteria for morbidity followed by haemorrhagic disorders (80.9 %) and other systemic disorders (38.3 %). In total, 83.0 and 72.3 % of the cases required blood transfusion and ICU admission, respectively. Among the 47 cases, 26 (55.3 %) women developed near miss conditions at arrival or within 12 h of arrival and 15 (31.9 %) developed the conditions 12 h after arrival; of these women, the number of referred cases were 16 (61.5 %) within 12 h of arrival and eight (53.3 %) 12 h after arrival. Three cases of uterine rupture were reported: one with an unscarred uterus and two with a scarred uterus.

The overall ICU admission rate was 0.3 % (67/21,579) and the admission rate for women with maternal near miss was 72.3 %. Haemorrhagic disorders constituted 78.9 % (abruptio placenta and ruptured uterus, 100.0 %; postpartum haemorrhage, 80.8 %; abnormal placental invasion, 80.0 %; ectopic pregnancy, 50.0 %) of all maternal near miss admissions to the ICU. In contrast, hypertensive disorders constituted 50.0 % (severe hypertension, 100.0 %; eclampsia , 50.0 %; severe pre-eclampsia, 42.9 %) and other systemic disorders constituted 72.2 % (shock, 100.0 %; thrombocytopenia, 76.9 %; seizures, 50.0 %). For hysterectomy, 17 of 19 cases (89.5 %). For hysterectomy, 17 of 19 cases (89.5 %) underwent caesarean section (14 emergency and three elective caesarean section) and 12 (63.2 %) had previous caesarean section.

Table 4 complements the information in Table 3 regarding the underlying causes of severe maternal morbidity and maternal near miss. Previous caesarean section contributed to most cases of severe maternal morbidity (22.5 %) and maternal near miss (36.2 %). Anaemia was a contributing factor in 17.2 % of severe maternal morbidity cases and in 27.7 % of maternal near miss cases (Table 4).

**Table 4:** Underlying causes of severe maternal morbidity and maternal near miss

Underlying causes	SMM (*n* = 395)	MNM (*n* = 47)
	*n* (%)	*n* (%)
Anaemia	68 (17.2)	13 (27.7)
Previous caesarean section	89 (22.5)	17 (36.2)
Prolonged/obstructed labour	11 (2.8)	2 (4.3)

*SMM* severe maternal morbidity, *MNM* maternal near miss

### Organ dysfunction criteria

Maternal near miss conditions identified according to organ system dysfunctions are shown in Table 5. The most common organ dysfunctions reported among maternal nearmiss cases were coagulation/haematologic dysfunction (74.5 %) followed by uterine (40.4 %) and cardiovascular (34.0 %) dysfunctions. Half (*n* = 25, 53.2 %) of the women with maternal near miss had one organ dysfunction; 12 (25.5 %) had two organ dysfunctions, eight (17.0 %) had three organ dysfunctions and two (4.3 %) had four organ dysfunctions.

**Table 5:** Maternal near miss conditions according to organ system dysfunctions

Organ dysfunction criteria	MNM (*n* = 47)
	*n* (%)
Clinical criteria	7 (14.9)
Acute cyanosis^b^	0 (0)
Gasping^b^	0 (0)
Respiratory rate >40 or <6/min^b^	1 (2.1)
Shock (SBP < 90 mmHg for >60/min and PR ≥ 120/min)^a^	4 (8.5)
Oliguria (urine output < 30 ml/h for 4 h or < 400 ml/24 h)^c^	0 (0)
Clotting failure^d^	0 (0)
Loss of consciousness lasting ≥ 12 h^f^	0 (0)
Loss of consciousness and absence of PR/HR^f^	1 (2.1)
Stroke ≥ 24h^f^	0 (0)
Uncontrollable fit^f^	0 (0)
Jaundice in the presence of pre-eclampsia^e^	0 (0)
Laboratory-based criteria	19 (40.4)
Severe hypoxaemia (oxygen saturation < 90 % for ≥ 60 min)^b^	1 (2.1)
Severe hypoxaemia (PaO_2_/FiO_2_ < 200 mmHg)^b^	0 (0)
Severe acidosis (pH < 7.1)^a^	3 (6.4)
Severe hypoperfusion (lactate > 5 mmol/l)^a^	10 (21.3)
Severe acute azotemia (creatinine ≥ 300 mmol/L or 3.5 mg/dl)^e^	0 (0)
Severe acute thrombocytopenia (< 50,000 platelets)^d^	9 (19.1)
Severe acute hyperbilirubinemia (bilirubin >100 mmol/L or > 6.0 mg/dl)^d^	0 (0)
Loss of consciousness and presence glucose and ketoacids in urine^f^	0 (0)
Management-based criteria	40 (85.1)
Used of continous vasoactive drug^a^	7 (14.9)
Intubation and ventilation ≥ 60 min not related to anesthesia^b^	6 (12.8)
Hysterectomy following infection or hemorrhage^g^	19 (40.4)
Dialysis for acute renal failure^c^	0 (0)
Transfusion of ≥ 5 units red cell^d^	29 (61.7)
Cardio-pulmonary resuscitation^a^	2 (4.3)
Organ dysfunction for the above conditions	47 (100.0)
Cardiovascular dysfunction	16 (34.0)
Respiratory dysfunction	8 (17.0)
Renal dysfunction	0 (0)
Coagulation/hematologic dysfunction	35 (74.5)
Hepatic dysfunction	1 (2.1)
Neurologic dysfunction	2 (4.3)
Uterine dysfunction	19 (40.4)

*MNM* maternal near miss; *SBP * systolic blood pressure; *PR* pulse rate; *HR *heart rate

^a^Cardiovascular dysfunction

^b^Respiratory dysfunction

^c^Renal dysfunction

^d^Coagulation/hematologic dysfunction

^e^Hepatic dysfunction

^f^Neurologic dysfunction

^g^Uterine dysfunction

Management-based criteria (85.1 %) were the most common criteria for maternal near miss followed by laboratory-based (40.4 %) and clinical (14.9 %) criteria. In total, 61.7 and 40.4 % of the cases required blood transfusion of ≥ 5 units of red blood cells and hysterectomy due to infection or haemorrhage, respectively. Severe hypoperfusion and severe acute thrombocytopenia were the most common laboratory findings. Most women presented with shock, unconsciousness and hypo- or hypertachypnoea on clinical examination (Table 5).

### End of pregnancy and pregnancy outcome

Table 6 shows the end of pregnancy and pregnancy outcome. One case presented at the emergency department with supra-pubic pain and was diagnosed as intra-abdominal bleeding following which exploratory laparotomy was performed. Placenta percreta was observed invading the urinary bladder; therefore, total hysterectomy and bladder repair were performed.

**Table 6:** End of pregnancy and pregnancy outcome

Variable	SMM (*n* = 395)	MNM (*n* = 47)
	*n* (%)	*n* (%)
End of pregnancy		
Mode of delivery		
Spontaneous vaginal delivery	64 (16.2)	6 (12.8)
Assisted vaginal delivery	13 (3.3)	1 (2.1)
Elective caesarean section	39 (9.9)	5 (10.6)
Emergency caesarean section	247 (62.5)	30 (63.8)
Laparotomy - ectopic pregnancy	12 (3.0)	3 (6.4)
Laparoscopy - ectopic pregnancy	19 (4.8)	1 (2.1)
Laparotomy - intra-abdominal bleeding	1 (0.3)	1 (2.1)
Term delivery		
Yes	262 (66.3)	28 (59.6)
No	133 (33.7)	19 (40.4)
Pregnancy outcome		
Foetal outcome		
Alive	350 (88.6)	38 (80.9)
Dead	45 (11.4)	9 (19.1)
Birth weight (kg)	2.8 (0.89)^a,b^	2.7 (0.91)^a,c^
Sex of baby		
Boy	183 (50.4)^b^	22 (52.4)^c^
Girl	179 (49.3)	19 (45.2)
Ambiguous	1 (0.3)	1 (2.4)
	SMM (*n* = 350)	MNM (*n* = 38)
	*n* (%)	*n* (%)
If alive		
Healthy	186 (53.1)	14 (36.8)
Admitted to neonatal ICU	168 (46.9)	24 (63.2)
	SMM (*n* = 45)	MNM (*n* = 9)
	*n * (%)	*n * (%)
If dead		
< 22 weeks gestation	32 (71.1)	5 (55.6)
Fresh stillbirth	6 (13.3)	2 (22.2)
Macerated stillbirth	3 (6.7)	2 (22.2)
Early neonatal death	4 (8.9)	0 (0.0)

*SMM* severe maternal morbidity, *MNM* maternal near miss

^a^mean (standard deviation)

^b^ *n* = 363. 32 women were at less than 22 weeks gestation

^c^ *n* = 42. Five women were at less than 22 weeks gestation

In total, 24 cases of fresh stillbirths and 153 cases of macerated stillbirths were reported at these two hospitals (*n* = 177). Abruptio placenta was the primary cause of death for the fresh stillbirths (five abruptio placenta, one sepsis) and macerated stillbirths (two abruptio placenta, one not known). Three of five fresh stillbirth abruptio placental cases and one of two macerated stillbirth abruptio placental cases were preterm. Early neonatal deaths were due to abruptio placenta (term deliveries) (*n* = 2) and foetal distress secondary to prolonged second stage labour (*n* = 2).

### Standard procedures and process indicators

Table 7 shows the process indicators related to specific conditions. For each condition, the target population was identified and the proportion of that target population receiving the recommended evidence-based intervention was examined. Higher proportions of women receiving appropriate interventions indicate better quality of care. The expected proportion of women receiving oxytocin for prevention and treatment of postpartum haemorrhage was almost 100.0 %. Two cases did not receive oxytocin. The first case was woman with pre-eclampsia who was found to have placenta accreta during emergency caesarean section; hysterectomy was performed. In the second case, emergency caesarean section was performed because of secondary arrest of labour. The patient developed severe pre-eclampsia following the operation.  Instead of oxytocin, intramuscular haemabate was administered.

**Table 7:** Process indicators

Process indicator	SMM		MNM	
	Target population	*n* (%)	Target population	*n* (%)
Prevention of postpartum haemorrhage^a^	363	361 (99.4)	42	41 (97.6)
Treatment of severe postpartum haemorrhage^b^	210	208 (99.0)	26	25 96.2)
Anticonvulsants for eclampsia^c^	22	22 (100.0)	2	2 (100.0)
Prevention of severe systemic infections or sepsis^d^	286	282 (98.6)	35	35 (100.0)
Treatment of severe infections and sepsis^e^	2	2 (100.0)	0	

*SMM* severe maternal morbidity, *MNM* maternal near miss

^a^The number of women who received a single dose of oxytocin for the prevention of postpartum haemorrhage divided by the number of all women giving birth

^b^The number of women with postpartum haemorrhage who received therapeutic oxytocin divided by the number of all women with postpartum haemorrhage

^c^The number of women with eclampsia who received magnesium sulfate divided by the number of all women with eclampsia

^d^The number of women having a caesarean section and receiving prophylactic antibiotics divided by the number of all women having caesarean sections

^e^The number of women with severe systemic infections or sepsis who received antibiotics divided by the number of all women with severe systemic infections or sepsis

## Discussion

Descriptively, women aged less than 35 years old, without history of previous caesarean sections or past pregnancy complications constituted higher proportions of severe maternal morbidity and maternal near miss cases. Different studies have reported the magnitude of severe maternal morbidity or maternal near miss as incidence or prevalence. However, incidence, referring to new cases, may not differ from all cases reported during the study period, i.e., prevalence, as both were directly related to the complications that occur in the index of pregnancy, labour or puerperium. The various measurements make interpreting and comparing the cases inaccurate [9]. Therefore, to harmonize the measurements, prevalence was used to report the findings in this study.

### Severe maternal morbidity

Severe maternal morbidity includes a broader category of women who suffered complications related to pregnancy, delivery and puerperium that were not necessarily associated with critical illness such as blood transfusion. The WHO criteria not only consider clinical disorders (haemorrhagic, hypertensive and other systemic disorders) but also severe management indicators to indicate the severity and to enhance the identification of severe maternal morbidity [3]. In this study, approximately 2 % (18.3 per 1000 live births) of the 21,756 study population had severe maternal morbidity, which is comparable with the proportions recently reported elsewhere (0.8 to 17.5 %) [7, 8, 10–15]. The primary causes of severe maternal morbidity were postpartum haemorrhage (53.2 %) and severe pre-eclampsia (26.1 %). Blood transfusion and ICU admission criteria represented 48.9 and 17.0 %, respectively, of women with severe maternal morbidity in this study.

### Maternal near miss

The WHO has proposed 25 criteria based on the presence of organ and system dysfunctions (cardiovascular, respiratory, renal, coagulation, hepatic, neurologic and uterine dysfunctions) via clinical, laboratory- and management-based parameters for identification of maternal near miss [3]. Even using similar proposed criteria, the studies published to date have reported considerable differences in the proportions of maternal near miss. Most studies have reported a maternal near miss prevalence ranging from 0.4 to 3.3 % [7, 8, 10–21]. Only one study in India reported an extremely high proportion of maternal near miss of 12.0 % based on a retrospective record review [22]. One large study, WHO Multicountry Survey on Maternal and Newborn Health (WHOMCS) reported the prevalence of maternal near miss of 0.8 % among 314,623 women attending 357 health facilities in 29 countries in Africa, Asia, Latin America and the Middle East (2538 maternal near miss and 486 maternal deaths) [23]. Twelve countries were involved in Asia, excluding Malaysia. Another recently published study in Nigeria also reported a prevalence that was within the reported range (1.6 %) [24].

The maternal near miss incidence ratio found in this study was 2.2 per 1000 live births (0.2 %) and was slightly low compared to other studies. As per the WHO criteria for maternal near miss, shock (8.5 %), severe hypoperfusion with lactate >5 mmol/L (21.3 %) and transfusion of five or more units of red blood cells (61.7 %) were the most common clinical, laboratory- and management-based parameters, respectively. Management-based parameters were the most frequently associated criteria for near miss (85.1 %) followed by laboratory-based criteria (40.4 %). This finding strengthens the specificity of management-based criteria in detecting severe obstetric cases for maternal near miss [25]. Postpartum haemorrhage, abnormal placental invasion and severe pre-eclampsia were the leading causes of maternal near miss (55.3, 21.3 and 14.9 %, respectively).

Considering the differences in healthcare systems and populations, the slightly lower prevalence of maternal near miss was justified. First, in this study, the women screened for organ and system dysfunction criteria for maternal near miss were from a pool of women initially classified with severe maternal morbidity. Although a slightly lower prevalence of maternal near miss was identified, the prevalence of severe maternal morbidity was within the reported range. Second, the data collection allowed clarification of doubts regarding the records obtained from the health personnel who took care of the women. This finding indicates that the data were real and unlikely due to underreporting. Steps were taken to ensure the data quality including pre-entry checking for completeness and double-checking medical records for unclear cases.

Third, ectopic pregnancy, which has a different aetiological pattern for maternal morbidity, accounted for approximately 8 % of maternal near miss cases similarly reported elsewhere [16, 21]. This figure is lower than that found in other studies (approximately 12 %) with high maternal near miss prevalence [13, 24]. Studies including a high number of early pregnancy losses in the numerator and using deliveries or live births as the denominator would inflate the prevalence of maternal near miss [26].

### Maternal death

Maternal deaths are tragic events in obstetrics; however, severe maternal morbidity was estimated to be 100 times more common than death [27]. Our study found that severe maternal morbidity was almost 200 times more common than death, with almost 400 episodes affecting approximately 22,000 women in these two tertiary centres. Maternal death indeed constitutes the most obvious manifestation of severe morbidity related to pregnancy, childbirth and puerperium.

Sixteen maternal deaths occurred in Kelantan during the study period, seven of which were due to direct causes; three, indirect causes; and six, fortuitous deaths. Direct maternal deaths are due to obstetric complications, whereas indirect deaths are due to previously existing diseases or from a disease that develops and is aggravated during pregnancy. The 10 maternal deaths were caused by haemorrhage (three cases), eclampsia (one case), ectopic pregnancy (one case), amniotic fluid embolism (one case), infection (two cases) and undetermined (two cases). The WHO estimates that haemorrhage accounted for 27.1 % of maternal deaths; hypertensive disorders, 14.0 %; and sepsis, 10.7 %. The other deaths were due to abortion (7.9 %), embolism (3.2 %) and all other direct causes of death (9.6 %) worldwide [28].

### Maternal near miss mortality ratio

In addition to prevalence, other indicators such as the maternal near miss mortality ratio and mortality index were used to describe the obstetric care. The maternal near miss mortality ratio estimates the complexity of care and refers to the ratio of maternal near miss cases and maternal death. This ratio also represents the proportion of maternal near miss cases that progressed to maternal death; the higher the ratio, the better the quality of care that the women received [4]. During the study period, two maternal deaths occurred and 47 cases of maternal near miss were identified in the two tertiary centres, revealing a maternal near miss mortality ratio of 23.5. Thus, for every 24 to 25 maternal near miss conditions, one maternal death occurred. The occurrence of at least four maternal near misses to every one maternal death also confirms other reports in the literature that justify the study of near miss cases [29].

Clearly, maternal near miss cases are more likely to die in resource-poor settings. For example, using similar WHO criteria [4], studies conducted in South Africa (Nigeria, Tanzania and Ghana) and Pakistan observed near miss to mortality ratios ranging from 1.5 to 2.5 [8, 17, 24, 30], while Brazil, India, Nepal and Iraq had near miss to mortality ratios ranging from 3.3 to 8.6 [7, 10–13, 15, 16, 18–20]. The ratio reported in this study is relatively high but is consistent with a study performed in China (23.0) [14] in which a similar method of data collection was applied. Higher ratios indicate a low maternal mortality ratio and better quality of care [4]. An overall increase in the ratio was observed in Brazilian studies with data collected from tertiary maternity hospitals from 2008 to 2012 [7, 10, 12, 16, 19, 20], except for a study by Lotufo et al*.* [15] that was conducted with data from a general intensive care unit. The increase in the near miss to mortality ratio over the period reflects an improvement in obstetric care. Therefore, instead of a single estimation, yearly estimations may facilitate monitoring and improving the quality of care provided.

### Mortality index

The mortality index is an indicator to represents an estimate of performance. This index refers to the number of maternal deaths divided by the number of women with maternal near miss and maternal death and is expressed as a percentage [4]. Our findings revealed a mortality index of 4.1 %, which is comparable to the 4.2 % mortality index reported by Shen et al*.* (2013) in China. A high index (>20 %) indicates low quality obstetric care for severe cases, in which more women with severe conditions die. In contrast, a low index (< 5 %) indicates better quality of care, with fewer women with severe conditions dying [4]. Thus, the health facilities in this study are performing well regarding management of complex and severe cases.

High mortality indexes ranging from 22.9 to 40.8 % were reported in several studies in Africa, Latin America and Pakistan [8, 17, 19, 20, 24, 30]. Of these studies, two were the early retrospective studies in Brazil (2008 to 2009) [19, 20] and the highest index was reported in Nigeria [24]. In contrast, other studies in Brazil, Iraq, India and Nepal reported indexes between 10.4 and 18.1 % [7, 10–13, 16, 18].

### Clinical disorders

Haemorrhagic and hypertensive disorders were the two leading causes of severe maternal morbidity and maternal near miss in our study. This finding is consistent with other published studies in developing countries [11, 18, 30]. These obstetric events, which correlate with death, were also identified as the leading causes of maternal death in Malaysia [2].

Haemorrhagic disorders, primarily postpartum haemorrhage, ectopic pregnancy and abruptio placenta, account for approximately 50, 8 and 6 % of the severe maternal morbidity cases, respectively. A similar pattern observed in the maternal near miss cases indicated the potential progression of haemorrhagic complications to near miss and death. The finding that postpartum haemorrhage contributes the largest proportion is in line with the findings of other severe maternal morbidity and maternal near miss studies (36.1 to 48.5 %) [14, 18, 30]. Most of the cases of postpartum haemorrhage were due to uterine atony, consistent with studies reported elsewhere [15, 31, 32].

Ectopic pregnancy occurs in 1 to 2 % of all pregnancies and is the primary cause of morbidity and mortality in the first trimester of pregnancy [33]. The occurrence of ectopic pregnancy was 3.2 % [34] of severe maternal morbidity cases and approximately 12 % [15, 24] of maternal near miss cases. In our study, the occurrence of ectopic pregnancy was approximately 8 % in both conditions. The prevention of ectopic pregnancy in severe maternal morbidity cases remains a challenge as no risk factors have been identified that can predict severe intra-abdominal bleeding [35].

Abruptio placenta, the single most important cause of antepartum haemorrhage, complicates approximately 0.4 to 1 % of pregnancies [36], accounting for 7.1 % of severe maternal morbidity cases and 6.4 % of maternal near miss cases in our study, within the range reported elsewhere [15, 24, 37].

Hypertensive disorders, specifically pre-eclampsia, were the second highest cause of morbidity in our study. In spite of the high proportion of pre-eclampsia cases, the relatively low proportion of eclampsia cases may suggest adequate prevention of seizures. The progression to organ dysfunction occurred in seven cases (6.8 %) of pre-eclampsia and in two cases (9.1 %) of eclampsia. Appropriate and timely obstetrical care such as administration of magnesium sulphate and delivery of the placenta is crucial for preventing of morbidity and mortality. Even so, pre-eclampsia, which was thought to be a self-limited entity, appears to cause real damage to cardiovascular endothelial function [38]. Hypertensive disorders were also strongly associated with an increased risk obstetric complications such as abruptio placenta, thrombocytopenia and pulmonary oedema [39, 40].

Fluid overload imposed on severe pre-eclampsia patients may increase the risk of acute pulmonary oedema. In our study, acute pulmonary edema was prevalent in 2.5 % of severe maternal morbidity cases and in 6.4 % of maternal near miss cases. A much lower occurrence (0.9 %) of severe maternal morbidity cases was observed [15]. Limited data were available for comparison based on the WHO criteria. Among the severe hypertensive disorders patients, maternal near miss cases and maternal deaths were unsurprisingly high (16.6 %) but only 0.8 % severe maternal morbidity cases were observed [39].

The risk of uterine rupture is a concern for patients with previous caesarean section delivery. A case-control study on uterine rupture by the intended mode of delivery using the United Kingdom Obstetric Surveillance System (UKOSS) reported that the risk of uterine rupture with planned vaginal delivery in women with prior caesarean section was low, at approximately one in 500 deliveries [41]. A subsequent UKOSS study showed that the risk was more importantly associated with prostaglandin induction and oxytocin treatment during labour [42]. Therefore, the use of prostaglandin and oxytocin should be cautiously used during labour to improve patient safety.

### Contributory factors

In our study, previous caesarean section was found in 22.5 and 36.2 % of women with severe maternal morbidity and maternal near miss, respectively, contributing to the vulnerable status of this population. This result is consistent with findings from other studies regarding severe maternal morbidity (9.5 to 34.9 %) [7, 8, 11, 14] and maternal near miss (18.1 to 56.7 %) [8, 10, 11]. Previous or current caesarean section is often associated with increased likelihood of hysterectomy [43], consistent with our study, which found that among the 19 women with hysterectomy, 17 underwent caesarean section (14 emergency and 3 elective) and 12 (63.2 %) had previous caesarean section. This result suggests increased anticipation towards postpartum haemorrhage and peripartum hysterectomy for cases with previous and current caesarean sections. However, the presence of anaemia was unlikely to contribute to the morbid condition.

### Management of severe cases

Haemorrhagic complications had great potential for progression into near miss, which is consistent with the high proportions of blood transfusion and hysterectomy in this study. Blood transfusion of five or more units of red blood cells indicates severe haemorrhage and a life-saving measure, whereas fewer units may indicate moderate anaemia to enhance postpartum recovery. A high proportion of large blood transfusion (61.7 %) was observed in our study. This result was much higher than proportion of blood transfusion (24.0 to 46.3 %) observed in maternal near miss cases in some studies [14, 15, 21]. Therefore, continuous adequate supplies and early access to blood products are crucial for successful rescue.

For patient safety, peripartum hysterectomy due to obstetric haemorrhage was commonly performed when other treatment modalities failed. In the same studies, in addition to receiving large blood transfusion, hysterectomy was performed [14, 15, 21]. All women that consented to hysterectomy (primarily for uterine atony, abnormal placental invasion or uterine rupture) in our study facilities survived. This finding highlights the need for an available surgical team to perform hysterectomy whenever required or an early referral to centres providing such services.

In our study, ICU admission with obstetric complications corresponded to 3.1 per 1000 live births. The most frequent obstetric morbidities transferred to ICU occurred following hysterectomy (17/19, 89.5 %) and haemorrhage due to placental implantation disorders (10/13, 76.9 %). The ICU transferals were for immediate post-operative surveillance and for intensive treatment purposes such as shock. The admission of obstetric cases to the ICU was very low, at 0.3 % (less than 0.5 %) and the admission of maternal near miss cases to the ICU was barely above the recommended standard, at 73.5 % (70 %) [4]. Approximately 20 to 50 % of women with maternal near miss failed to receive ICU care from events such as postpartum haemorrhage, placental implantation disorders and eclampsia, corroborating with the substantial proportion (46.8 %) of women experiencing two and more organ dysfunctions. This result suggests a shortage of ICU beds or the need to review the number of readily available ICU beds for obstetric patients.

Women presenting with obstetric complications may require a higher level of care. Those women with organ dysfunction would be more appropriately managed in the ICU to provide optimum care and to minimize the number of multiple organ failures [44]. Although an evidence-based triage system to assist clinicians regarding maternal utilization of intensive care services is lacking, haemorrhagic and hypertensive disorders were the two most common disorders in which admission into the ICU was deemed necessary [45].

### Organ dysfunction

Characterization of maternal near miss conditions according to organ system dysfunction is unquestionably feasible in tertiary hospitals in which procedures for monitoring are routinely performed without resource constraints [46]. By occurrence, the coagulation/haematologic dysfunction was the most common event followed by uterine and cardiovascular dysfunctions in our study. These events were manifested by large blood transfusion, hysterectomy and severe hypoperfusion criteria.

Cases that developed complications on arrival or within 12 h of arrival must be separated from those cases that developed complications in the hospital setting because the former would indicate failure in accessing tertiary centres and/or referral systems. Approximately half of women with severe maternal morbidity (46.3 %) and maternal near miss (55.3 %) developed complications within 12 h of admission, of which 68.3 and 61.5 % were referred cases, which may partly indicate an issue of delay in the referral system. Second, interestingly, the mortality index for in-hospital patients was 5.0 % compared to 3.4 % for referred cases. The higher mortality index suggests that the quality of care provided to in-hospital patients needs to be further reviewed concerning clinical management.

### Essential interventions

The use of oxytocin for the prevention and treatment of postpartum haemorrhage, of magnesium sulphate for the treatment of eclampsia, of prophylactic antibiotics for caesarean section and of parenteral antibiotics for the treatment of sepsis was covered almost 100 %. The coverage of recommended interventions below 95 % should be interpreted as an opportunity to improve care [4]. Good adherence to the measurable standards of the WHO guidelines suggests good quality of care in these facilities.

High coverage of essential interventions was observed in various countries participating in the WHOMCS; 18 % of women with severe maternal outcomes (maternal near miss and maternal death) did not receive the indicated interventions [23]. In poor-resource settings, a lack of information regarding organ dysfunction and an inadequate assessment of severity may contribute to the suboptimal implementation of essential interventions and clinical management. However, in our study, the process indicators of basic interventions seemed to be widely practiced and did not provide additional information regarding tertiary hospitals.

Coverage of essential interventions was suggested as the first important step in analysing the issues related to the quality of care. However, notably, application of these single elements of care in the provision of a comprehensive care system may not be adequate [23, 28]. For an instance, in relation to the care for postpartum haemorrhage, apart from the use of oxytocin for its prevention and treatment, other aspects of care such as shock management, an adequate donated blood supply and prompt surgical interventions are essential. Similarly, for the care of eclampsia, the role of magnesium sulphate is fundamental but severe hypertension management, pre-delivery stabilization and airway restoration are also vital. Although our study fared better with respect to coverage of essential interventions, further reduction of maternal mortality can be achieved by addressing these deficiencies.

The relevance of the essential interventions in further reducing maternal mortality has recently been questioned [47]. For example, in the WHOMCS, first, the risk of death did not increased [*OR* (95 % CI): 1.3 (0.81, 1.97], *P* = 0.330] in women with severe maternal outcomes that missed the opportunity to receive essential interventions compared to women without missed opportunities. Second, paradoxical performance regarding maternal mortality was observed. Coverage of all the five essential interventions was low in countries with low MMR and high in countries with high MMR. Although the differences between countries with low, moderate, high and very high MMR were not significant for three of the essential interventions, the coverage were significantly different for prophylactic antibiotics for caesarean section and parenteral antibiotics for sepsis. Thus, elements of care other than the essential interventions play an important role in the survival of women with morbid conditions [23].

According to the WHO process indicator, women presented at the hospital with obstructed labour or uterine rupture should undergo operations within three hours of admission. Failure to do so reflects substandard care and suggests an intra-hospital delay in the management of obstructed labour [4]. In our study, the cases of ruptured uterus occurred beyond the time limit. However, this process indicator is limited to cases of obstructed labour and uterine rupture presented at the hospital and not for hospitalized patients, suggesting the need for an improved indicator of ruptured uterus or new indicators related to prolonged or obstructed labour.

### End of pregnancy and pregnancy outcome

In our study, more than half of the pregnancies were terminated via emergency caesarean section, indicating the urgency of the obstetric conditions. Babies delivered to mothers with severe maternal morbidity and maternal near miss require neonatal ICU admission more frequently, as similarly reported by Lotufo et al*.* [15]. Half of the indications for admission to the neonatal ICU in our study were linked to preterm birth. Stillbirths due to severe maternal morbidity contributed to 5.1 % of all stillbirths, while, abruptio placenta accounted for 77.8 % of stillbirths in severe maternal morbidity cases. Among cases with abruptio placenta, the perinatal mortality was 32.1 %. In addition to the severity of abruptio placenta, foetal survival depends on the gestational age [36], and most of the stillbirths occurred prematurely, between 25 and 35 weeks.

### Recommendation

Notably, the causes of severe maternal morbidity and maternal near miss were similar to the leading causes of maternal death. This result is relevant for identifying interventions that can be performed during antenatal care and delivery to reduce maternal death. Auditing of these cases demonstrated to be feasible despite the large number of cases [48]. In addition, the process of auditing would lead to increased experience in tackling the determinants of maternal morbidity [48] and understanding the real demands at each level of health care [3].

Obstetric emergencies, namely, haemorrhagic and hypertensive disorders should remain the priority topics for training, although the stagnant progress in reducing maternal mortality may not be attributed to the lack of knowledge regarding its complications and management. Of note, the possible reasons for this lack of progress may lie in the organization, delivery and utilization of services, as explained by the disproportionately small number of maternal deaths (2/10, 20.0 %) occurring in tertiary centres compared to lower level health facilities, with most of the complications having developed during the course of labour and delivery. Implementing a sur**v**eillance strategy for women with severe maternal morbidity allows early identification of complications and better preparedness for acute morbidities. This implementation includes ongoing evidence-based guidelines, audits, obstetric table top simulations and awareness of red flags among junior health care providers or lower level facilities to minimize delays in management and referrals.

Cases of ruptured uterus occurring in hospitalized women are an important indication of the seriousness of substandard care. The current process indicator has outlined that women presented at the hospital with obstructed labour or uterine rupture should undergo operation within three hours of admission. Modification and improvement of this indicator is highly required. This study suggests for the monitoring to begin from the time these diagnoses were made for hospitalized patients but with shorter time limit. 

Of 10 maternal deaths, two deaths occurred in tertiary centres and four deaths occurred in hospitals with and without obstetrics and gynaecology specialists (the remaining four women were brought in dead). This result implies that, apart from patient factors, the occurrence of severe maternal morbidity and maternal near miss, similar to maternal deaths, may be influenced by the health care level. Research involving different levels of health facilities should be repeated to objectively identify issues related to pregnancy complications upon which appropriate interventions could be adopted. Apart from allowing comparisons between health facilities, a single health facility could monitor its progress over time.

### Limitations

The findings of this study should not be regarded as representative of Kelantan but indicative of a large hospital-based tertiary centre study. The maternal outcomes in community-based birthing centres and district level hospitals were not assessed. Utilization of the WHO maternal near miss criteria seems to be limited in smaller health facilities. The laboratory-based (for example, pH, PaO_2_ and lactate) and management-based markers (for example, vasoactive drug management and hysterectomy) are less likely to be applicable in these health facilities. Therefore, we support severe maternal morbidity as the initial classification in the continuum that begins with any occurrence of complications of pregnancy, delivery and puerperium until death. It is proposed that this pragmatic criteria approach related to severe maternal morbidity be applied at poor-resource health facilities and the strict near miss criteria approach related to organ dysfunctions be applied for quantitative assessments and international comparisons. Thus, the strict near miss criteria should be incorporated as much as possible [49]. These approaches should complement each other to obtain information to improve maternal health.

## Conclusion

The reduced maternal morbidities corresponded with the high coverage of essential interventions in these tertiary centres. Comprehensive emergency care and intensive care and overall improvements in the quality care for maternal health need to be further examined to achieve substantial reductions in maternal death.

## Abbreviations

CEMD: confidential enquiry into maternal deaths
ICU: intensive care unit
LSCS: lower segment caesarean section
MDG: Millennium Development Goals
MMR: maternal mortality ratio
MR: Malaysian Ringgit
*OR*: odds ratio
UKOSS: United Kingdom Obstetric Surveillance System
WHO: World Health Organization
WHOMCS: WHO Multicountry Survey on Maternal and Newborn Health
